# Erratum to: “Gas chromatography-mass spectrometry in the taxonomy of Miscanthus”

**DOI:** 10.18699/VJ20.603

**Published:** 2020-02

**Authors:** N.M. Slynko, N.V. Burmakina, O.M. Potseluyev, S.Yu. Kapustyanchik, G.Yu. Galitsin, T.N. Goryachkovskaya, L.V. Kuybida, S.V. Shekhovtsov, S.E. Peltek, V.K. Shumny

**Keywords:** 10.18699/VJ19.583, 10.18699/VJ19.583

## Abstract

Vavilovskii Zhurnal Genetiki i Selektsii = Vavilov Journal of Genetics and Breeding. 2019;23(8):1076-1081 (in Russian) Page 1081, in Acknowledgements instead of This work was supported by State Budgeted Project АААА-А17-117092070032-4. should read This work was supported by State Budgeted Project 0259-2019-0011. The original article can be found under DOI 10.18699/VJ19.583

## Abstract (ru)

Вавиловский журнал генетики и селекции. 2019;23(8):1076-1081 На странице 1081 в разделе Благодарности вместо «Работа поддержана бюджетным проектом № АААА-А17-117092070032-4». следует читать: «Работа поддержана бюджетным проектом № 0259-2019-0011». Оригинальная статья может быть найдена по DOI 10.18699/VJ19.583

